# The Fifth Bioelectronic Medicine Summit Hosted by the Feinstein Institutes for Medical Research (Manhasset, NY) and Columbia Engineering (NY, NY) at The Garden City Hotel, Garden City, NY 11530 on October 11-12^th^, 2002- Bioelectronic Medicine: Today’s Tools, Tomorrow’s Therapies: Sponsored by IRIS Biomedical, Neuromodec, Bioelectronic Medicine (journal), and Feinstein Institutes for Medical Research/Northwell Health

**DOI:** 10.1186/s42234-023-00105-6

**Published:** 2023-03-14

## Poster session abstracts

### P1. Clinical feasibility of liver-targeted peripheral ultrasound neuromodulation (pFUS) using interleaved B-mode imaging and pulsed ultrasound stimuli in type 2 diabetics

#### Jeffrey Ashe^1^, John Graf^1^, Radhika Madhavan^1^, Kirk Wallace^1^, Victoria Cotero^1^, Samantha Abate^1^, Ram Krishna Pandey^1^, Raimund Herzog^2^, Porindla Sridhar Narayan^1^, David Shoudy^1^, Ying Fan^1^, Tzu-Jen Kao^1^, Chris Puleo^1^

##### ^1^ GE Research, Niskayuna, NY, USA; ^2^ Yale University, New Haven, CT, USA

###### **Correspondence:** Chris Puleo

Recently it has been shown that mechanical shear waves produced by focused ultrasound pulses result in activation of mechanosensitive ion channels and modulate peripheral nerve activity. The activity of multiple classes of ion channels can be affected by the pulsed ultrasound stimuli, and these ion channels show cell and tissue specific expression and activation patterns. Unlike electromagnetic stimuli, ultrasound penetrates deeply through biological tissue and can be focused to millimeter-to-centimeter-sized volumes without affecting neighboring tissue. Furthermore, ultrasound pulses can be non-invasively image-targeted to specific anatomical locations with technologies used safely for decades in diagnostic imaging applications. However, while peripheral neuromodulation with ultrasound has been demonstrated in multiple in vitro and pre-clinical models, the methods have not yet been reported in humans. Herein, we demonstrate the use of a modified diagnostic imaging system for testing pulsed ultrasound neuromodulation in human subjects. Specifically, we test pulsed ultrasound stimulation of the hepatoportal plexus using stimulation parameters previously found to restore glucose homeostasis and improve insulin resistance in multiple pre-clinical models of type 2 diabetes. We demonstrate image-targeted neuromodulation by interleaving B-mode imaging pulses with focused pulsed stimuli and apply both human expert-based and post-hoc deep learning-based image object detection to quantify the on-target versus off-target stimulus dose (i.e., the duration of time and spatial average intensity of pulses that were aligned to the anatomical target). Finally, we report the first safety and feasibility outcomes in subjects with type 2 diabetes and discuss these outcomes in relation to our previous pre-clinical results.

### P3. Toward closed-loop bioelectronics: wireless power and data communication using magnetoelectric technology

#### Fatima Alrashadan^1^, Zhanghao Yu^1^, Joshua Woods^1^, Kaiyuan Yang^1^, Jacob T. Robinson^1,2,3^

##### ^1^Department of Electrical and Computer Engineering, Rice University, Houston, Texas, USA; ^2^Department of Bioengineering, Rice University, Houston, Texas, USA; ^3^Department of Neuroscience, Baylor College of Medicine, Houston, Texas, USA

###### **Correspondence:** Jacob T. Robinson

Implantable bioelectronics has opened up a promising avenue to treat many drug-resistance neurological and psychiatric disorders by interfering directly with the nervous system. These devices deliver controlled electric stimulations to modulate the electrical activities of the nervous system or record the electrical, chemical, and physical properties for better diagnosis. Integrating implantable devices with wireless power, bidirectional communication, and biosensing functionalities could allow for the design of next-generation bioelectronics that features adaptive closed-loop systems and distributed networks of implants. Different wireless power transfer techniques (WPT) including radiofrequency (RF), inductive coupling, ultrasound, and light have demonstrated the ability to power medical implants, however, with performance trade-offs in terms of implant size, misalignment tolerance, bidirectional communication link, and the amount of power that can be delivered safely through biological tissues.

Magnetoelectric (ME) is an emerging technology that has shown the potential to wirelessly power miniaturized implants (ME-BIT) to stimulate different nerve targets while demonstrating high efficiency at mm-size, high power delivery (> 1 mW) without safety issues, and high misalignment tolerance. These features are empowered by using ME material that has high power density, low mechanical resonance frequency, and high permeability to concentrate magnetic flux inside the material. However, to enable using the ME technology to design adaptive closed-loop systems, there is still a need to develop a bidirectional data communication link. One possible solution is to integrate the ME implant with one of the existing communication modalities, including, RF, near field coupling, ultrasound, or infrared, however, the addition of new components will increase the device footprint and constrain the power budget.

Here, we propose a low-power communication system that utilizes the unique characteristic of the magnetoelectric material itself to establish a bidirectional link between the ME-BIT and an external transceiver. Specifically, we use the backscattered field generated by the ME film when excited by an external magnetic field as a carrier signal. To modulate this signal to encode digital data, we use an integrated circuit to change the electrical loading conditions across the ME film terminals. This carrier signal travels losslessly through the biological tissues and can be received using a magnetic receiver outside the body.

Our system comprises an implantable ME-BIT (ME transducer + IC) and a custom portable transceiver. With design optimizations in data modulation and recovery, the proposed system archives an 8-kbps data rate at the 335-kHz carrier frequency, and a TRX-implant distance greater than 2 cm for a bit error rate less than 1E-3. Furthermore, we validated the proposed system for wireless stimulation and sensing and conducted Ex-vivo tests through a 1.5-cm porcine tissue.

### P4. High frequency electrical stimulation attenuates neuronal release of high mobility group box 1 (HMGB1) and ameliorates neuropathic pain

#### Huan Yang^1^, Timir B. Datta-Chaudhuri^1,2,3^, Sam J. George^1^, Bilal Haider^1^, Jason Wong^1^ Michael Brines^1^, Kevin J. Tracey^1,2,3^, and Sangeeta S. Chavan^1,2,3^

##### ^1^Institute for Bioelectronic Medicine, Feinstein Institutes for Medical Research, 350 Community Drive, Manhasset, NY 11030, USA; ^2^Elmezzi Graduate School of Molecular Medicine, Feinstein Institute for Medical Research, Northwell Health, Manhasset, NY, USA; ^3^Donald and Barbara Zucker School of Medicine at Hofstra/Northwell, Hempstead, New York, USA

###### **Correspondence:** Sangeeta S. Chavan

Chronic pain presents a major unmet clinical problem. High-frequency electrical nerve stimulation (HFES) has achieved clinical success as an analgesic modality for pain management, but the underlying mechanism is unknown. We reasoned that HFES may inhibit neuroinflammatory mediator release by sensory neurons to reduce pain. HMGB1, a key mediator of injury- and infection-elicited inflammation, is involved in the pathogenesis of persistent pain. We recently reported that neuronal HMGB1 is required for mediating inflammation and hyperalgesia following nerve injury (Yang H et al., PNAS, 118:1-9, 2021). Here we assessed the effects of HFES in modulating HMGB1 release by sensory neurons. Using microelectrode arrays (MEAs) in cultured dorsal root ganglia (DRG) harvested from transgenic mice that express light-sensitive channel rhodopsin in sensory neurons, we observe that light-evoked HMGB1 release from DRGs is significantly reduced with HFES exposure (10 kHz, 2 mA, rectangular symmetric, biphasic, charge balanced) (HMGB1 levels in unstimulated group = 5.3 + 0.5 ng/ml; in light stimulated group = 25.8 + 6.0 vs. light + HFES = 8.2 + 2.1* pg/ml, N=6, *: P<0.01 vs. light stimulated group). In agreement, in vivo studies showed that HFES (20.6 kHz, 10 min/per day X 3 consecutive days) significantly reduces mechanical hyperalgesia and levels of HMGB1 in the inflamed paws in C57BL/6 mice that subjected to chronic constriction injury (CCI) of the sciatic nerve. HMGB1 levels in sham surgery group = 10.6 + 1.1 ng/mg protein, in CCI group = 29.0 + 3.9 vs. CCI + HFES = 12.8 + 1.6* ng/mg protein, N=10 mice per group, *P<0.001 vs. CCI group. Similar beneficial effects by HFES were observed in Sprague-Dawley rats subjected to sciatic nerve injury. Together, these results support the mechanistic insight that HFES may reset sensory neurons into a less pro-inflammatory state via inhibiting the release of neuroinflammatory mediators such as HMGB1 (supported in part by funding from TrueRelief, Santa Monica, CA; grants from NIH, NIGMS 1R35GM118182 to KJT and R01GM132672 to SSC).

### P5. Solid state batteries enable miniaturisation of active implanted medical devices

#### Denis Pasero

##### ^1^Ilika, Unit 10a The Quadrangle, Abbey Park Industrial Estate, Romsey, UK

###### Correspondence: Denis Pasero

Purpose: A new generation of miniaturised, implanted, active medical devices, possibly introduced via a catheter, is being developed by disruptive product designers. Conventional medical batteries are packaged within metallic cans for safety purposes; they are also typically primary (non-rechargeable) and must contain the whole energy required during the life of the device they power from first day of implantation. For these reasons, miniaturisation of conventional medical batteries is limited to a few 10’s of cm3. New active implanted sensing devices are being designed with less than 1 cm3 in volume, including implanted cardiac sensors, neuromodulation therapy devices, smart orthopaedics and orthodontics sensors. Millimeter-scale solid state batteries, which do not need significant packaging, have been developed to uniquely enable miniaturisation of next generation implantable devices.

Methods: Solid state batteries were fabricated by physical vapour deposition and sputtering. Key developments to increase energy density used the following methods:

- Implement photolithography as a method for patterning the batteries sub-layers at the micron-level, enabling miniature features
- Thin down the substrate, i.e. the mechanical support for the batteries, enabling high energy density
- Stack and interconnect single cells on top of each other, to multiply the energy of the resulting battery for a same footprint
- Increase the cathode thickness in order to store more energy

The rechargeable batteries have been developed on Ilika’s first volume manufacturing line, opened in Southampton, Hampshire in 2021, the first of its kind in the UK.

Results: Ilika’s first solid state batteries were produced, down to 15 mm2 footprint and total thickness 1 mm. The batteries consisted of 6 stacked cells, interconnected in parallel, yielding a total capacity of 300 uAh and nominal voltage of 3.5V. The arial energy density of the stacked battery was measured to be approximately 12.5 uAh/mm2. Internal resistance of the full stack was measured to be about a sixth that of each single cell forming the stack, enabling peak power of a few mA. These batteries could be recharged in as little as 8 min with heating of the battery less than 2°C upon fast charging. These batteries are going through their final development stage and will go through full medical certification in 2023.

Discussion: A novel technique for stacking and interconnecting solid state cells was shown to significantly increase the energy density and decrease the internal resistance of the battery stack. This development could enable further development in implanted medical sensors by providing an energy source of minimal size (mm-scale footprint and um-scale thickness), appropriate energy density for increasing functionalities and long life avoiding the risk and cost of removal.

Use of rechargeable batteries for in-the-body applications have historically suffered from patient compliance with regards to regular charging. Whilst a new conversation with the patient is required, the benefit of miniaturising non-life-critical sensors which can be recharged in less than 10 minutes, is expected to outweigh the need for regular recharging.

### P7. Flexible, scalable high channel count stereo-electrode for recording in the human brain

#### Keundong Lee,^1^ Angelique C. Paulk,^2^ Yun Goo Ro,^1^ Karen Tonsfeldt,^1^ Youngbin Tchoe,^1^ Andrew M. Bourhis,^1^ Jihwan Lee,^1^ Daniel R. Cleary,^1^ John S. Pezaris,^2^ Yoav Kfir,^2^ Sydney S. Cash,^2^ Shadi A. Dayeh^1^

##### ^1^Integrated Electronics and Biointerfaces Laboratory, Department of Electrical and Computer Engineering, University of California San Diego, La Jolla, CA 92093, USA; ^2^Department of Neurology, Harvard Medical School, Boston, MA, USA; Center for Neurotechnology and Neurorecovery, Department of Neurology, Massachusetts General Hospital, Boston, MA 02114, USA

###### **Correspondence:** Shadi A. Dayeh

Over the past decade, stereotactically placed electrodes have become the gold standard for deep brain recording and stimulation for a wide variety of neurological and psychiatric diseases. Current devices, however, are highly limited in their spatial resolution and ability to record from small populations let alone individual neurons. Here, we report on a novel, reconfigurable, monolithically integrated human-grade flexible depth electrode with a maximum of 128 channels and able to record from 10 cm of brain tissue. This thin, stylet-guided depth electrode is capable of recording local field potentials and single unit neuronal activity (action potentials) as validated across species and represents a major new advance in manufacturing and design approaches for a mainstay technology in clinical neurology.

### P8. NINDS and trans-NIH funding opportunities for technology development and translation

#### Eric Hudak, Megan Frankowski, Brooks Gross, Nick Langhals

##### Division of Translational Research, National Institute of Neurological Disorders and Stroke, National Institutes of Health, Bethesda, Maryland, USA

###### **Correspondence:** Eric Hudak

The mission of the NINDS Division of Translational Research (DTR) is to accelerate basic research findings toward patient use for neurological disorders and stroke by providing funding, expertise, and resources to the research community. DTR provides funding and resources through grants, cooperative agreements, and contracts to academic and industry researchers to advance early-stage neurological technologies, devices, and therapeutic approaches to industry adoption (i.e. investor funding and corporate partnerships).

The Translational Neural Devices (TND) program within DTR has created a variety of programs that support the design, implementation, and management of research activities critical to translational challenges in the treatment of neurological disease. In addition, TND plays an active role in the NIH BRAIN Initiative, Blueprint MedTech Program, SPARC Program, and HEAL Initiative.

NINDS TND is actively managing programs that support neural device therapeutics and training through grants, contracts, and consultants. These programs cover all stages of translational research from early device development and optimization to preclinical development and early clinical development. Funding opportunities and resources are actively supporting translational research in preclinical discovery and development of new therapeutic interventions for neurological disorders and stroke, as well as neuropsychiatric disorders and neurotraumatic injuries (BRAIN Initiative) and pain (HEAL Initiative). An overview of the NINDS Translational Neural Devices program and related Trans-NIH funding opportunities and resources will be presented.

### P9. Bioelectronic devices for in vivo recordings of metabolic information from the vagus nerve

#### Amparo Guemes, Poppy Oldroyd, Alejandro Carnicer-Lombarte, Sam Hilton, George Malliaras

##### Bioelectronic Laboratory, Department of Engineering, University of Cambridge, Cambridge, UK

###### **Correspondence:** Amparo Guemes

Introduction: Closed-loop bioelectronic medicine, which uses electrical recording and stimulation to interface with peripheral nerves, is becoming a promising strategy for the treatment of chronic diseases [1]. This multidisciplinary work presents the opportunities of bioelectronics for improving metabolic control in Type 1 diabetes by decoding metabolic clues from the cervical vagus nerve recordings.

Methods: Interfacing with peripheral nerves and organs for reliable recording poses difficult challenges due to invasiveness and high risk of body rejection. Here, we compare the ability of two types of sub-millimetre cuff devices to record metabolic information from the vagus nerve: thin-film flexible devices were chosen given their potential for chronic implantation, and gold microwires were used as gold-standard. Both type of devices were implanted in vivo around the cervical vagus nerve of Sprague-Dawley rats anaesthetised under urethane. For the microwires, a region of 1mm was exposed towards the tip of PTFE coated gold microwires (75um diameter) and placed around the nerve for cuff recordings. Conformable bioelectronic devices were fabricated using thin-film technology based on conductive polymer PEDOT:PSS microelectrodes on parylene-C for high-resolution recording [2]. The devices include two ring microelectrodes to cover the circumference of the vagus nerve and 6 small contacts (100umx100um) to increase spatial resolution. In this work, we also present an improved strategy to extract information from neurograms under different metabolic conditions. The decoding methodology applies band-pass filtering (400Hz-4Khz), ECG removal based on continuous wavelet transformation (CWT), identification of action potentials and extraction of waveforms, dimensionality reduction, clustering of waveforms, extraction of metrics (spike rate, spike amplitude, inter-spike-interval), and correlation with metabolic events.

Results: We have validated the ability to record nerve activity and decode metabolic information through implantation of both gold microwires and flexible devices on the vagus nerve of rodents after metabolic challenges. Using both types of devices, we predominantly found an increase in the overall firing rate or at least in one of the identified clusters corresponding to a decrease in glucose levels. No meaningful changes in the firing rate were observed on increasing or stable blood glucose concentrations. To verify that the detected spikes were of neural nature, a lidocaine bath at the exposed site was used at the end of the experiments that caused the successful elimination of the neural response.

Conclusion: We demonstrate the design, fabrication, and acute implementation of new bioelectronic neural probes for recording signals from the surface of the vagus nerve. Gold microwires can be used as a gold-standard electrophysiological tool for acute recording from the vagus nerve of rats under anaesthesia, but bioelectronic flexible thin-film devices based on conductive polymers provide the best way for minimally invasive chronic implantation in awake animals. This study explores the development of new technology to interface with small peripheral nerves, and contributes to increase our understanding on the role the nervous systems plays in metabolism.

[1] Guemes, A., Etienne-Cummings, R., and Georgiou, P. Bioelectronic Medicine 6(1). (2020).

[2] D. Khodagholy, et al., Adv. Mat. 2011, vol 23, H268-H272.

### P10. A system for investigating bioelectronic therapies using a multicontact nerve cuff electrode and an implanted stimulator/recorder

#### Joern Rickert^1^, Martin Schuettler^1^, Ivor Gillbe^2^, Ronny Pfeifer^1^, Timothy Denison^3^

##### ^1^CorTec, Freiburg, Germany; ^2^Bioinduction Ltd., Bristol, UK; ^3^Institute of Biomedical Engineering, Department of Engineering Sciences, University of Oxford, Oxford, UK; MRC Brain Network Dynamics Unit, Nuffield Department of Clinical Neurosciences, University of Oxford, Oxford, UK

###### **Correspondence:** Timothy Denison

Introduction: The field of bioelectronic medicine has moved quickly in the past years from fundamental research on rodents to first clinical studies, e.g., for treatment of rheumatic arthritis or hypertension. New applications are on the horizon but the transfer from fundamental research to clinical studies requires hardware suitable for large animal or human use, which is not commonly available today. Here, we describe a platform suitable for investigating new biomedical treatment paradigms in large animals and humans.

Methods and Materials: The platform combines the Picostim ultracompact stimulator/recorder implant system by Bioinduction (Bristol, UK), the DyNeuMo research application developed in partnership between Bioinduction and Oxford, and the AirRay electrode technology developed by CorTec (Freiburg, Germany).

Results: The Picostim implant is powered by a rechargeable battery and features 8 channels of electrical stimulation (up to 15mA), neural recording, and a 3-axis accelerometer. The implant is very compact at just 7cc and is programmed remotely using an external wireless controller. The implant allows smart adaptive therapy employing circadian, motion and neural signal feedback, which are combined and processed in real time on the implant.

The nerve interface consists of a novel self-sizing spiral nerve cuff electrode with 8 contacts. Two of the contacts are ring shaped, arranged at the ends of the cuff. The remaining 6 contacts are dot-shaped and describe a segmented ring in the middle of the cuff. The cuff is 18mm in length and has an inner diameter of 2.5mm. It is made from laser-micromachined soft silicone (PMDS) and platinum iridium contacts. The cuffs are attached to multi-lumen polyurethane-based cables/connectors.

Conclusions: Combined, the platform can be used to develop new and more precise treatments using recording and fascicle selective stimulation on the vagus nerve as well as other nerves. Currently, preclinical versions of the system are undergoing tests and validations, and clinical versions are planned. Here, we present the technical details of the system and its application**.**

### P11. Non-invasive 40Hz sensory stimulation as a potential novel therapeutic intervention for neurodegenerative diseases

#### Mihály Hajós^1,2^, Aylin Cimenser^1^, Xia Da^1^, Alyssa Boasso^1^, Chandran V. Seshagiri^1^, Holly Mrozak^1^, Evan Hempel^1^, J. Thomas Megerian^1,3^, Brent Vaughan^1^, Zach Malchano^1^

##### ^1^Cognito Therapeutics, Cambridge, MA, USA; ^2^Yale University School of Medicine, New Haven, CT, USA; ^3^Thompson Autism Center, CHOC Children’s, Orange, CA, USA

###### **Correspondence:** Mihály Hajós

Recent experimental findings have shown that synchronized 40 Hz gamma oscillation of neuronal networks, generated by optogenetic or sensory (e.g., visual and/or auditory) stimulation, effectively reduce pathological hallmarks of Alzheimer’s disease (AD) in transgenic mice expressing AD-related human pathological genes (Adaikkan & Tsai, 2020). Daily one-hour exposure to 40Hz visual and auditory stimulation showed reductions in brain AD plaques, hyperphosphorylated tau, neurodegeneration and brain atrophy. Treatment also prevented synaptic loss and dysfunction, leading to improved learning abilities in transgenic mice. These results initiated the development and validation of non-invasive, 40Hz sensory stimulation as a potential therapeutic intervention for AD and potentially other neurodegenerative diseases. In a 6-month long phase I/II randomized, controlled, US-based multi-center clinical trial (Overture trial; NCT03556280) the feasibility, safety, tolerability, adherence rates and efficacy of gamma sensory stimulation were evaluated using Cognito Therapeutics Gamma Sensory Stimulation System in patients with clinical presentation of AD spectrum. Participants with Mini-Mental State Examination (MMSE) scores of 14-26, were randomized 2:1 to receive daily, one-hour, 40Hz noninvasive audio-visual stimulation or sham stimulation.

A total of 135 subjects were screened, of whom 74 were randomized and 53 completed (20 sham arm, 33 active arm). Daily use of the Gamma Sensory Stimulation System was confirmed to be safe with minimal side effects. MRI data demonstrated absence of Amyloid-Related Imaging Abnormalities (ARIA) in all subjects. High adherence to daily therapy was established based on device-recorded usage. Among clinical instruments assessing cognitive and functional abilities, Alzheimer's Disease Cooperative Study - Activities of Daily Living (ADCS-ADL) and MMSE scores demonstrated the most effective outcomes of the therapy. Changes in ADCS-ADL scores were statistically significant between the sham and treatment arms (P<0.0003), indicating a significant slowing in functional decline by the treatment. Similarly, participants in the active arm showed a significantly reduced decline in MMSE scores compared to sham arm subjects (p<0.013). Nighttime active durations (assessed by actigraphy) were significantly (p<0.03) reduced in the treatment arm and the opposite change was observed in the sham arm. Quantitative MRI analysis revealed a significantly reduced whole brain (p<0.01), and occipital lobe (p<0.03) volume loss, and a significantly attenuated reduction in occipital cortical thickness (p<0.01) in the active arm relative to the sham arm participants. A separate MRI analysis revealed a significant (p<0.004) preservation of white matter in active treatment arm participants compared to ADNI1 controls.

Our results demonstrate that long-term, daily 40Hz sensory stimulation is safe and well tolerated. Based on the treatment’s excellent safety profile, together with beneficial clinical outcome results observed in the Overture trial, pivotal clinical trials have been planned to support clinical efficacy of gamma sensory stimulation in AD. In addition, based on significant reduction in white matter loss and brain atrophy, effects of gamma sensory stimulation should be explored in other neurodegenerative diseases as well.

### P13. Evoked synaptic excitatory potentials (ESAPs): a novel electrophysiological biomarker for spinal cord stimulation

#### Mahima Sharma^1^, Vividha Bhaskar^1^, Lillian Yang^1^, Nigel Gebodh^1^, Tianhe Zhang^2^, Rosana Esteller^2^, John Martin^1^, Marom Bikson^1^

##### ^1^Department of Biomedical Engineering, The City College of New York, NY, USA; ^2^Boston Scientific Neuromodulation, Valencia, California, USA

###### **Correspondence:** Marom Bikson

Introduction: Spinal cord stimulation (SCS) produces varied responses evoked by epidural electrical stimulation. Evoked responses occurring within 2 ms of stimulation are the electrically evoked compound action potentials (ECAP) that measure the activity of dorsal column axons but not necessarily a spinal circuit response. We identify a separate electrophysiological signal that occur 2-3 ms after SCS that directly reflects synaptic activity in the spinal grey matter.

Methods: Anesthetized female Sprague Dawley rats (250-280g) were implanted with epidural spinal cord stimulation (SCS) leads, epidural motor cortex stimulation electrodes, epidural recoding lead, and intra-spinal penetrating recording electrode arrays, as well as electromyography (EMG) electrodes. We stimulated motor cortex or the epidural spinal cord with a train of 20 pulses (200- 40 μs pulse width, 1 Hz) and simultaneously recorded responses from epidural and intraspinal electrodes.

Results: Low frequency (1 Hz) SCS produces characteristic ECAP (composed of P1, N1, and P2 waves lasting <2 ms) as well as an additional S-wave starting after the N2 and lasting ~6 ms. The S-wave has a distinct dose response and spinal topography compared to the ECAPs. CNQX (a highly selective competitive antagonist of AMPA) blocked the S-wave and its intraspinal analog but not ECAPS. Given its distinct synaptic origin, we term S-wave response an Evoked Synaptic Excitatory Potential (ESAP). Increasing SCS frequency to 50 Hz dampened ESAPs but not ECAPs, suggesting habituation.

Conclusions: We recorded a sparsely, if ever, characterized evoked spinal synaptic response (ESAPs) in addition to dorsal column ECAPs that may shed insight into SCS underlying mechanisms.

### P15. Focused ultrasound stimulation at the spleen does not produce hemodynamic changes and does not elicit antidromic compound action potentials in the splenic nerve

#### Stefanos Zafeiropoulos^1,2^, Naveen Jayaprakash^1^, Stavros Zanos^1,2^

##### ^1^ Elmezzi Graduate School of Molecular Medicine at Northwell Health, 350 Community Drive, Manhasset, NY 11030 USA; ^2^ Institute for Bioelectronic Medicine, Feinstein Institutes for Medical Research at Northwell Health, 350 Community Drive, Manhasset, NY 11030 USA

###### **Correspondence:** Stavros Zanos

Background: Focused ultrasound stimulation (FUS) of nerve terminals at the spleen has anti-inflammatory effects (Cotero, 2019) and, for that reason, it may have therapeutic value in various diseases (Zachs2019, Ahmed 2020). However, its mechanism of action is still unclear. Splenic nerve stimulation elicits compound action potentials (CAPs) in the splenic nerve and transiently increases systemic arterial blood pressure (Donega, 2021). It is unknown if splenic FUS produces hemodynamic effects or elicits antidromic CAPs in the splenic nerve.

Methods: Healthy rats (n=4) were anesthetized and FUS of the spleen was applied for 12 minutes at 0.83 MPa. Before, during and after FUS delivery, we measured right ventricular pressure, systemic blood pressure, and heart rate. At the same timepoints, we recorded neural activity from the splenic nerve; an i.v. LPS injection (10 Î¼g) was performed as positive control to validate splenic nerve recordings, as LPS is known to activate the splenic nerve (MacNeil, 1996).

Results: Right ventricular systolic pressure remained stable over the time course of the experiment (pre-FUS: 31.38 ± 1.35 mmHg, during FUS: 31.39 ± 1.17, post-FUS: 31.35 ± 1.35). Similarly, mean arterial pressure (pre-FUS 88.10 ± 1.95 mmHg, during FUS: 86.35 ± 1.66, post-FUS: 86.42 ± 2.71) and heart rate (pre-FUS: 254.89 ± 10.65, during FUS: 255.66 ± 12.43, post-FUS: 254.71 ± 12.9) did not change significantly. The rate of spontaneous CAPs recorded from the splenic nerve did not change with FUS (baseline: 0.28 ± 0.25 spikes/s, during FUS: 0.25 ± 0.22; paired t-test p= 0.46). Notably, spontaneous CAPs were elicited after LPS injection (0.51 ± 0.33 vs. 0.35 ± 0.30 with saline injection, aired t-test =0.03).

Conclusions: Splenic FUS in healthy rats does not affect arterial pressure in the systemic or pulmonary circulation, suggesting that it is hemodynamically safe. It does not elicit recordable antidromic CAPs in the splenic nerve, suggesting that its mechanism of action may include subthreshold depolarizations of nerve terminals without affecting nerve firing.

### P18. High intensity focused ultrasound stimulation of spleen reduces traumatic bleeding in mice

#### Carlos E. Bravo-Iñiguez^1,2^, Sangeeta S. Chavan^1,2,3^, Kevin J. Tracey^1,2,3^, Jared M. Huston^2,3,4^

##### ^1^Elmezzi Graduate School of Molecular Medicine, The Feinstein Institutes for Medical Research at Northwell Health, 350 Community Drive, Manhasset, NY 11030 USA; ^2^Institute of Bioelectronic Medicine, The Feinstein Institutes for Medical Research at Northwell Health, 350 Community Drive, Manhasset, NY 11030 USA; ^3^Department of Science Education, Donald and Barbara Zucker School of Medicine at Hofstra/Northwell, 500 Hofstra Boulevard, Hempstead, NY 11549 USA; ^4^Department of Surgery, Northwell Health, 300 Community Drive, Manhasset, NY 11030 USA

###### **Correspondence:** Jared M. Huston

Introduction: Uncontrolled hemorrhage is the most common preventable cause of traumatic death. Direct pressure or tourniquets limit extremity bleeding, but non-compressible hemorrhage requires surgical control. Systemic hemostatic therapies are limited. In preclinical models of hemorrhage, electrical vagus nerve stimulation (VNS) accelerates thrombosis and decreases traumatic hemorrhage in a spleen-dependent manner. Here we demonstrate that non-invasive high intensity focused ultrasound (HIFU) stimulation targeting the spleen significantly reduces bleeding in a murine tail transection model through CD4^+^ ChAT^+^ T lymphocytes and α7 nicotinic acetylcholine receptors (α7nAChR).

Methods: Adult male C57BL/6J mice, α7nAChR-deficient knockout mice, and conditional CD4^+^ ChAT^+^ T lymphocyte knockout (CD4-ChAT^fl/fl^) mice were anesthetized (ketamine/xylazine), placed in the right lateral decubitus position, and shaved after identifying the spleen by anatomical markers and palpation. The ultrasound probe was directed at the splenic hilum, followed by 1 min stimulation (1.1 MHz, 200 mV/pulse, 150 burst cycles, 500 s burst period), a 30 s rest, and 1 min stimulation for a 5 min total stimulation. Animal tails were warmed in water (37°C, 5 min), transected 2 mm from the tip, and bled into water until hemorrhage stops for a minimum of 10 s to record the duration of bleeding (bleeding time).

Results: Compared with sham stimulation, HIFU significantly reduces bleeding time following tail transection (Sham = 110.5 ± 7.7 s vs. HIFU = 72.9 ± 6.6 s, Mean ± SEM, n = 10/group, p < 0.01 (two-tailed t-test). Genetic deletion of α7nAChR (α7KO) or CD4^+^ ChAT^+^ T lymphocytes abolishes vagus nerve-mediated effect of HIFU on bleeding time (α7KO Sham = 125.7 ± 9.2 s vs. α7KO HIFU = 127.4 ± 9.5 s, Mean ± SEM, n = 8/group, p=0.5781 (two-tailed t-test), CD4-ChAT^fl/fl^ Sham = 141.0 ± 11.3s vs. CD4-ChAT^fl/fl^ HIFU = 128.3 ± 14.9 s, Mean ± SEM, n = 5 or 6/group, p=0.53 (two-tailed t-test).

Conclusions: These data demonstrate that HIFU stimulation of the spleen provides a novel method to reduce traumatic hemorrhage. HIFU warrants additional study in traumatic and surgical bleeding, and as a treatment for bleeding disorders.

### P19. Specific neuronal populations encode inflammatory responses

#### Okito Hashimoto^1^, Tyler Hepler^1^, Tea Tsaava^1^, Aisling Tynan ^1^, Kevin J. Tracey^1, 2^, Sangeeta S. Chavan^1,2^

##### ^1^The Feinstein Institutes for Medical Research, Northwell Health, Manhasset, USA; ^2^Donald and Barbara Zucker School of Medicine at Hofstra/Northwell, Hempstead, USA

###### **Correspondence:** Sangeeta S. Chavan

The immune system is regulated by the nervous system with principles of reflex regulation. Neuronal pathways, including the vagus nerve-based inflammatory reflex, are physiological regulators of immune function and inflammation. However, it remains unclear whether and how the brain encodes the state of the immune system. We previously showed that the projections from the brainstem dorsal motor nucleus of the vagus nerve control inflammatory responses in the spleen. Furthermore, we found that cytokine-specific information is present in sensory neural signals within the vagus nerve. Based on these findings, we reasoned that brain encodes and stores cytokine-specific information. Here, we carried out activity-dependent cell labeling in mice. We utilized the targeted-recombination-in-active-populations (TRAP2) mice crossed with a tdTomato reporter line to produce double transgenic TRAP2/tdTomato mice. We captured neuronal ensembles that were active in response to tumor necrosis factor (TNF) or interleukin-1β(IL-1β) administration. Increased tdTomato expression (indicative of neuronal activity) in response to TNF or IL-1β administration is observed in the paraventricular nucleus (PVN), the bed nuclei of the stria terminalis (BNST) and the nucleus of the solitary tract (NTS), where vagal afferents ascending from peripheral tissues synapse on neurons. As readout of systemic TNF or IL-1β administration, we investigated physiological parameters and serum level of interleukin-6 (IL-6) and monocyte chemoattractant protein-1 (MCP-1) that are induced by TNF and IL-1β. Increased serum level of IL-6 and MCP-1 is observed after both of TNF and IL-1 administration. Furthermore, interestingly, we found that IL-1β administration causes increase of heart rate, but TNF does not. These results suggest that there are specific neuronal populations in the brain regions activated by TNF or IL-1β, controlling different efferent pathways.

### P20. Stimulation of the brainstem dorsal motor nucleus of the vagus or the nucleus ambiguus produces distinct outcomes in lipopolysaccharide-induced endotoxemia

#### Aidan Falvey^1^, Santoshi. P. Palandira^1,2^, Kevin. J. Tracey^1,2,3^, Valentin. A. Pavlov^1,2,3^

##### ^1^Institute of Bioelectronic Medicine, Feinstein Institutes for Medical Research, Northwell Health, 350 Community Drive, Manhasset, NY 11030, USA; ^2^ Elmezzi Graduate School of Molecular Medicine, 350 Community Drive, Manhasset, NY 11030, USA; ^3^ Donald and Barbara Zucker School of Medicine at Hofstra/Northwell, 500 Hofstra University, Hempstead, New York 11549, USA

###### **Correspondence:** Valentin A. Pavlov

The vagus nerve regulates physiological processes including immunity and inflammation. The brainstem dorsal motor nucleus of the vagus (DMN) and nucleus ambiguus (NA) supply the motor (efferent) cholinergic neurons in the vagus nerve. Optogenetic DMN stimulation was recently shown to mitigate serum TNF levels during endotoxemia. The immunoregulatory effects of electrical DMN stimulation, however, remained unknown. Further, the contribution of the NA to the vagus nerve inflammatory regulation was unexplored. To provide insight, we investigated the effects of DMN electrical stimulation and NA electrical and optogenetic stimulation in mice with endotoxemia. Male mice (C57BL/6: Electrical & ChAT-ChR2-eYFP: Optogenetic stimulation) were anesthetized and fitted into a stereotaxic frame. A concentric bipolar electrode or a fiber-optic cannula was guided to the coordinates of the left DMN or left NA. Electrical stimulation (or sham stimulation) was performed for 5 mins and LPS (0.5 mg/kg) was injected (i.p.). 90 mins later blood and spleen were collected and processed for cytokine analyses. Optogenetic stimulation was utilized solely for the left NA and the endotoxemia challenge was similarly performed. Electrical DMN stimulation significantly reduced pro-inflammatory cytokine levels in the serum (TNF: p = 0.0012; IL-6: p = 0.427) and spleen (TNF: p = 0.0001; IL-6: p = 0.0077), as well as increasing the serum levels of the anti-inflammatory cytokine IL-10 (p = 0.0284) during endotoxemia. In contrast, neither optogenetic nor electrical NA stimulation altered serum and spleen cytokine levels during endotoxemia. In conclusion, these results indicate that while electrical DMN stimulation alters cytokine levels in endotoxemia: Stimulation of the NA (optogenetic or electrical) does not have significant effects. These findings identify the selective nature of efferent vagal neurons controlling cytokine responses and inflammation, which indicates future studies on brainstem nuclei, as potential targets for novel therapeutic strategies, should focus on the DMN, not the NA, for treatment of inflammatory conditions.

### P21. Assessing stability of spontaneous compound action potentials in vagus nerve recordings in a chronic implant mouse model

#### Shubham Debnath^1,2^, Ibrahim T. Mughrabi^1^, Todd J. Levy^1,2^, Fylaktis Fylaktou^1,2,3^, Yousef Al-Abed^1,3^, Stavros Zanos^1,3^, Theodoros P. Zanos^1,2,3^

##### ^1^Institute of Bioelectronic Medicine, Feinstein Institutes for Medical Research, Northwell Health, Manhasset, NY 11030, USA; ^2^Institute of Health System Science, Feinstein Institutes for Medical Research, Northwell Health, Manhasset, NY 11030, USA; ^3^Donald and Barbara Zucker School of Medicine at Hofstra/Northwell, Northwell Health, Hempstead, NY 11549, USA

###### **Correspondence:** Theodoros P. Zanos

The nervous system monitors and responds to physiological changes to maintain homeostasis. While the field of bioelectronic medicine has focused on modulating neural reflexes to treat diseases that affect autonomic responses to stimulation, less attention has been given towards resolving spontaneous activity of these circuits. The vague nerve (VN), one of the main conduits of these reflexes, has been a common target for bioelectronic devices. Recent studies have linked afferent sensory neuron activity in VN with inflammatory, metabolic, and cardiopulmonary biomarkers. However, most previous work has been based on terminal experiments, and no chronic recording studies have been performed in a mouse model, the preclinical model of choice for disease pathophysiology, genetic screenings, and pharmacological mechanism. In order to utilize sensory signals to diagnose emerging conditions or evaluate treatment efficacy, VN signals must be reliably recorded chronically and decoded to identify biomarkers.

A chronically implanted murine model has been developed and validated to deliver functional stimulation for at least four weeks (Mughrabi et al., 2021) and recording capabilities of this model have been reported (Debnath et al., 2021). The aforementioned model was used to record VN activity from anesthetized mice during repeated experiments across multiple days in the same animal, while simultaneously recording electrocardiography and nasal air flow to monitor heart and breathing rates. Each recording experiment involved 5-minute periods at three levels of isoflurane anesthesia (1.5%, 1%, and 0.5% of 1 L/min O2), after which VN stimulation was used to confirm viability of the electrode-nerve interface by establishing the heart rate threshold (HRT) needed to induce heart rate changes. Compound action potentials (CAPs) were extracted and sorted from raw neural recordings (Zanos et al., 2018).

Recordings were completed over multiple days, ranging from 1 to 185 days after implant in N=17 animals. To assess stability of CAPs across recording sessions/days from each animal, a composite similarity score was derived from computed correlations between various CAP features. These features are average waveform shape, firing rates and their changes due to isoflurane level, inter-CAP interval histograms, and phase-locking histograms on respiratory and cardiac cycles. This composite similarity score, along with the individual feature correlations, was computed across all recording days for each animal, and the resulting values were used to determine the number of unique CAPs and duration of continuous detection of each unique CAP. Signal-to-noise ratios (SNRs) and HRTs were also tracked and related to stability of CAPs.

Our study details both the methodology and results establishing stability of chronic recordings in murine models. These methods enable longitudinal analysis of spontaneous CAP recordings in the same animal and provide experimental and analytical frameworks for future studies. Future work will examine long-term changes in the VN recordings and CAP activity in response to induced disease states, as well as facilitate advancement of responsive closed-loop VN neuromodulation approaches.

### P22. The dynamic relationship of brain activity and heart rate variability during sympathetic and parasympathetic dominance in healthy individuals

#### Fylaktis Fylaktou^1,2,3^, Shubham Debnath^1,2^, Yousef Al-Abed^1,3^, Theodoros P. Zanos^1,2,3^

##### ^1^Institute of Bioelectronic Medicine, Feinstein Institutes for Medical Research, Northwell Health, Manhasset, NY 11030, USA; ^2^Institute of Health System Science, Feinstein Institutes for Medical Research, Northwell Health, Manhasset, NY 11030, USA; ^3^Donald and Barbara Zucker School of Medicine at Hofstra/Northwell, Northwell Health, Hempstead, NY 11549, USA

###### **Correspondence:** Theodoros P. Zanos

Background: The brain and the heart, through their dynamical interplay, ensure homeostasis, and mediate several physiological functions, and their disease-related aberrations, through the autonomic nervous system (ANS). ANS dysregulation, characterized by an imbalance between the sympathetic and parasympathetic arms, occurs in a plethora of conditions and its function is assessed in disease-specific test batteries. While multiple studies have aimed at characterizing the relationship between brain and heart function individually, to the ANS, few studies have attempted to examine interactions between the brain and the cardiovascular system during sympathetic or parasympathetic activation. We attempted to examine the interplay of heart and brain function, as reflected by heart rate variability (HRV) and electroencephalography (EEG) metrics measured non-invasively, while driving ANS function to sympathetic or parasympathetic dominance, using standardized, clinically relevant tests, in healthy, able-bodied humans.

Methods: Twenty-one individuals were assessed in multiple sessions. Each session included four standard clinical tests probing autonomic function: squat test, diving reflex test, deep breathing, and Valsalva maneuver. Noninvasive sensors captured continuous electrocardiography (ECG), and EEGs. From the ECGs, both temporal (root mean square of successive differences - RMSSD, standard deviation of NN intervals -SDNN, Poincare derived 2nd standard deviation) and spectral (Low Frequency (LF) Power, High Frequency (HF) Power, LF/HF ratio) HRV metrics were calculated and averaged across participants for each test. For the EEGs, the power of five frequency bands (delta (0.5-4Hz), theta (4-8 Hz), alpha (8-14 Hz), beta (14-30Hz) and gamma (30-100Hz)) were calculated. Power estimates were averaged across electrodes grouped spatially according to their anatomical locations (left or right, frontal, parietal, temporal and occipital) and the resulting power signals were averaged across participants for each test. Cross correlations, with a maximum lag of ±180sec were calculated for all possible combinations for EEG bands, electrode groups and HRV metrics. For each comparison, we captured both the time lag and the value of the maximum correlation coefficient and established statistical significance, Bonferonni corrected for multiple comparisons.

Results: Out of 960 comparisons, 25 comparisons yielded statistically significant correlations (p<0.00005), further divided into 7 strong (0.4-0.6), 5 medium (0.3-0.4) and 13 weak correlations of HRV and EEG power. Out of 4 autonomic tests, the deep breathing (parasympathetic) and squat (sympathetic) test revealed significant EEG-HRV correlations, and out of 6 different HRV metrics, only the LF/HF ratio had significant correlations with EEG power. In the parasympathetic test, strongest correlations were found between theta and alpha power of left temporal EEG electrodes, and LF/HF ratio, with time lags of 60-94 seconds, indicating HRV leads changes in EEG power. During the squat test, strongest correlations were found between alpha and gamma power of bilateral occipital and parietal EEG electrodes, with time lags of 6-9 seconds, indicating EEG power leads HRV changes.

Conclusion: Our results reveal aspects of the dynamic heart brain interplay, during sympathetic or parasympathetic dominance in healthy individuals. Our approach could be used potentially for diagnosis, measurement of disease progression, or treatment evaluation.

### P23. Chronic vagus nerve stimulation in mice induces distinct splenic immune responses in a stimulation schedule-dependent manner

#### Ibrahim Mughrabi^1†,^ Izumi Kurata-Sato^2†^, Michael Gerber^1,3,^ Yousef Al-Abed^1^, Stavros Zanos^1,3,^ Betty Diamond^2,3^

##### ^1^ Institute of Bioelectronic Medicine, The Feinstein Institutes for Medical Research, Manhasset, NY, USA; ^2^ Institute of Molecular Medicine, The Feinstein Institutes for Medical Research, Manhasset, NY, USA; ^3^ Zucker School of Medicine at Hofstra Northwell, Hempstead, NY, USA † These authors contributed equally

###### **Correspondence:** Betty Diamond

Acute vagus nerve stimulation (VNS) alters the innate and adaptive immune response in the spleen; however, the effects of chronic vagus nerve activation on the splenic immune response have been inferred from studies of post-sepsis mice. To directly assess the effects of chronic VNS on the immune response, healthy mice were implanted with a left cervical VNS device and received awake stimulation throughout the day/night cycle for 4 weeks following one of three stimulation schedules: brief and frequent (10 sec on, 5 min off), long and frequent (5 min on, 1 hour off), and long and infrequent (5 min twice a day) trains, using 500 μs pulses at 30 Hz pulsing frequency. To probe B and T cell responses, mice were immunized with a T-dependent antigen on week 2 and sacrificed on week 4 of chronic VNS. All groups exhibited a reduction in high-affinity serum IgG titers compared to sham-stimulated controls, whereas only frequent stimulus trains reduced low-affinity and total IgG titers, irrespective of train duration. Furthermore, chronic VNS increased the frequency of splenic FoxP3^+^ CD4^+^ regulatory T cells (Tregs), most notably with frequent trains, without affecting total CD4^+^ T cell counts. Splenic B220^+^ B cell frequency only decreased with long stimulus trains, irrespective of train frequency. These data indicate that chronic VNS produces distinct B and T cell responses in the spleens of stimulated mice, and those responses depend on the daily VNS schedule.

### P24. Inflammatory disease alters cytokine-specific signaling in vagus nerve sensory neurons

#### Tomas S. Huerta^1^, Bilal Haider^1^, Richard Adamovich-Zeitlin^1^, Adrian C. Chen^1^, Saher Chaudhry^1^, Sangeeta S. Chavan^1,2^, Kevin J. Tracey^1,2^, Eric H. Chang^1,2^

##### ^1^ Forman Family Laboratory of Biomedical Science, Institute of Bioelectronic Medicine, Feinstein Institutes for Medical Research, Manhasset, NY, USA; ^2^ Zucker School of Medicine at Hofstra/Northwell, Hofstra University, Hempstead, NY, USA

###### **Correspondence:** Eric H. Chang

Inflammatory bowel disease (IBD) is a chronic gastrointestinal disorder that includes Crohn’s disease and ulcerative colitis. IBD has a high financial burden and many patients become treatment-resistant to therapies including anti-TNF biologics. Peripheral neuropathy is a frequently reported complication of IBD, which suggests that the pathophysiology of IBD may involve the nervous system. To study a potential neural involvement in IBD, we focus on the vagus nerve (VN), which highly innervates the gastrointestinal tract, regulates gut function, and senses metabolites. Further, the VN is a component of the inflammatory reflex, a homeostatic pathway for the resolution of inflammation. Here, we used the dextran sodium sulfate (DSS)-induced colitis model of IBD and studied the effect of chronic inflammation on VN sensory neuron activity through in vivo calcium imaging of the nodose ganglion. Additionally, proinflammatory cytokines were administered directly on the VN to study individual sensory neuron responses to immune mediators.

This study utilized calcium imaging on transgenic VGLUT2-GCaMP6f mice. Using a Python-based calcium imaging analysis software, CaImAn, the change in fluorescence over baseline fluorescence (DFF) was plotted as calcium transients that correspond to the activity of individual neurons. To establish colitis, experimental mice were given 4% DSS in their drinking water and monitored for up to 8 days. In vivo calcium imaging was performed when mice had peak disease activity. The spontaneous activity of neurons was monitored during baseline, as well as the responses to cytokine administration directly on the VN.

Four % DSS lead to a robust colitis phenotype with the DSS group having significantly increase disease activity score compared to control mice (P < 0.001, mixed-effects ANOVA). Calcium imaging revealed that the DSS group had significantly increase spontaneous activity in VN sensory neurons (P < 0.05, Mann-Whitney U test). Notably, the peak amplitude of the calcium transients in the DSS group was significantly reduced when compared to controls (P < 0.01, Mann-Whitney U test). Direct administration of cytokines on the VN induced distinct responses specific to the cytokine applied. Comparison between TNF and IL1β revealed that TNF responses had on average higher peak amplitude (P < 0.001, Mann-Whitney U test), longer duration (P < 0.001, Mann-Whitney U test), larger integral of response (P < 0.01, Mann-Whitney U test) and a smaller number of peaks (P < 0.05, Mann-Whitney U test). Finally, when cytokines were applied directly on the VN of DSS mice, TNF responses were significantly reduced in terms of peak amplitude (P < 0.01, Mann-Whitney U test), and number of peaks (P < 0.05, Mann-Whitney U test) compared to control mice. Conversely, no significant changes were found in IL1β responses.

Our findings suggest that chronic inflammation results in robust changes in the activity of individual vagus nerve sensory neurons. Cytokines were found to generate specific responses in the nodose ganglion neurons, with the colitis condition impacting TNF-specific neural responses in DSS mice but not IL1β specific responses. These results implicate chronic inflammation as a potential driver for sensory neuron adaptation, a novel mechanism that warrants further investigation.

### P25. Human-grade high channel count ECoG and SEEG electrodes for recording (4096ch) and stimulation (256ch)

#### Jihwan Lee,^1^ Youngbin Tchoe,^1^ Keundong Lee,^1^ Angelique Paulk,^2^ Sydney Cash,^2^ Sharona Ben-Haim,^3^ Ahmed Raslan,^4^ Shadi A. Dayeh^1^

##### ^1^Integrated Electronics and Biointerfaces Laboratory, Department of Electrical and Computer Engineering, University of California San Diego, La Jolla, CA 92093, USA; ^2^Department of Neurology, Harvard Medical School, Boston, MA, USA; Center for Neurotechnology and Neurorecovery, Department of Neurology, Massachusetts General Hospital, Boston, MA 02114, USA; ^3^Department of Neurological Surgery, University of California San Diego, La Jolla, CA 92093, USA; ^4^Department of Neurological Surgery, Oregon Health and Science University, Portland, OR 97239, USA

###### **Correspondence:** Shadi A. Dayeh

Epilepsy is one of the most prevalent neurological diseases worldwide and over a quarter of all epilepsy patients experience drug-resistant epilepsy [1] where surgical interventions or electrical stimulation paradigm become most effective paradigms for treatment. The ECoG (electrocorticography) and SEEG (Stereoelectroencephalography) are the gold-standard mapping techniques to identify epileptogenic zones and to delineate boundaries between pathological and healthy tissue. High spatiotemporal resolution with a broad cortical coverage is crucial for precise localizations to enhance our understanding of epilepsy, improve patient outcomes and reduce functional impairments and potentially side effects. However, current clinical ECoG and SEEG electrodes have low spatial resolution and channel count where individual contacts are from 2 to 3 mm in diameter and up to 10 mm in intercontact spacing for ECoG and 6 mm for SEEG.

Building upon our first-in-human brain mapping with 1024-channel microelectrode array on pathological tissues in an acute intraoperative setting, [2] we have designed and scaled ECoG grids to 6.4 x 6.4 cm2 area with 4096 recording channels and 256 stimulation channels. Novel platinum nanorods (PtNRs) were utilized as electrode contacts for low impedance, high charge injection capacity, and long-term stability and parylene C as the insulating material for transparency and conformality to brain surface and movements. By utilizing advanced multi-layer parylene C fabrication methods with a total device thickness of 15 μm, large 6.4 x 6.4 cm^2 area with 1 mm electrode spacing was achieved with 30 μm diameter recording contacts and 1 mm diameter stimulation contacts. Both recording and stimulation contacts achieved over 90% yield, with average impedance of 20 kΩ and 800 kΩ, respectively.

Furthermore, the multi-layered fabrication approach allowed development of ultra high-density ECoG grids with 1 x 1 mm^2 area and 1024 channels with 20 μm diameter PtNR contacts, spaced 31 μm apart. The flexibility of parylene C also allowed wrapping of the high-density grids around a polyurethane tube for usage as a stylet-guided SEEG probe. Both record-breaking, ultra high-density ECoG grids and SEEG electrodes were tested in pilot whisker-barrel experiments in rats. Multi-layer ECoG grid functionality was demonstrated through placement of such electrodes on the rat primary somatosensory (SI) whisker barrel cortex and recording stimulus-evoked activity in response to air-puff stimulation onto whisker pad.

Overall, our results advance the scaling and development of high-channel clinical grids for semichronic epilepsy monitoring platform with fully wireless data and power transfer, and also paves the way toward other applications in responsive neurostimulator systems and brain-machine interfaces.

### P26. Spatially-selective cervical vagus nerve stimulation produces differential organ-specific responses

#### Weiguo Song^1^, Khaled Qanud^1^, Jacquelyn-Nicole Tomaio^1^, Naveen Jayaprakash^1^, Stavros Zanos^1,2^

##### ^1^Institute of Bioelectronic Medicine, Feinstein Institutes for Medical Research, Manhasset, NY, USA; ^2^Zucker School of Medicine at Hofstra/Northwell, Hempstead, NY, USA

###### **Correspondence:** Stavros Zanos

Vagus nerve stimulation (VNS) electrically activates vagal fibers innervating different organs to treat organ conditions. VNS is typically delivered in a non-selective manner, resulting oftentimes in reduced efficacy and side effects from organs that are not the primary target. The vagus nerve in swine and in humans has a fascicular structure; at the cervical level, fascicles in the vagal trunk are spatially arranged according to the organ to which they project. We developed a 10-contact cuff electrode that accommodates the fascicular organization of the cervical vagus nerve to test whether spatially-selective VNS can produce organ-specific stimulation responses. In anesthetized swine, spatially-selective VNS produces physiological responses from different organs that depend on the radial location on the nerve of the stimulated contact, resulting in radially asymmetric responses. Those responses include laryngeal muscle contraction, changes in breathing, changes in heart rate, and changes in blood pressure. In addition, spatially-selective VNS elicits radially asymmetric stimulus-evoked compound action potentials (eCAPs) from fibers with different conduction velocities, corresponding to distinct morphological fiber types. The angular separations of organ-specific responses agree with those of fiber-specific eCAPs and with the spatial arrangement of organ-specific fascicles in the cervical vagus. In awake swine, spatially-selective VNS elicits radially asymmetric cough reflex responses, with angular distributions that remain consistent for several weeks. We conclude that spatially-selective cervical VNS through a multi-contact cuff electrode device produces differential responses from innervated organs depending on the stimulated contact and can potentially be exploited for organ-specific vagus neuromodulation.

### P27. Differential physiological responses to cytokines IL-1b and TNF

#### Tyler D. Hepler^1^, Okito Hashimoto^1^, Tea Tsaava^1^, Aisling Tynan^1^, Kevin J. Tracey^1,2,3^, Sangeeta S. Chavan^1,2,3^

##### ^1^Institute for Bioelectronic Medicine, The Feinstein Institutes for Medical Research, Northwell Health, NY, USA; ^2^Donald and Barbara Zucker School of Medicine at Hofstra/Northwell, Hempstead, USA; ^3^The Elmezzi Graduate School of Molecular Medicine, Northwell Health, Manhasset, NY, USA

###### **Correspondence:** Sangeeta S. Chavan

The proinflammatory cytokines, interleukin-1β(IL-1β) and tumor necrosis factor (TNF) have been implicated to play a major role in driving sickness behavior, including lethargy, depression, anorexia, fever, and cognitive impairment. However, differential physiological changes specific to IL-1β and TNF are understudied. Here we show that IL-1β and TNF play a differential role in maintaining thermoregulation and locomotion. We monitored body temperature and activity in conscious and unrestrained mice by radiotelemetry using probes implanted into the peritoneal cavity. Intraperitoneal administration of IL-1β (Baseline IL-1β: 38.23 ±0.35, Post-Injection IL-1β: 36.35ï ±0.63) or TNF (Baseline TNF-α: 37.76 ±0.16, Post-Injection TNF-α: 36.67 ±0.15) induces a significant change in body temperature in mice compared to saline group (Baseline Saline: 38.00 ±0.21, Post-Injection Saline: 37.70 ±0.23). Administration of IL-1β (IL-1β: p=0.0019, Min BTIL-1β: 34.01 ±0.4752) but not TNF (TNF: Min BTTNF-α: 35.73 ±0.1937) induces a significantly lower minimum body temperature compared to saline injected mice (Saline: Min BTSaline: 36.50 ±0.1760) in the two hours following injection. Analysis of locomotive responses across a 6-hour (360 minute) window following cytokine administration revealed a significant decrease in activity in response to TNF in conscious and unrestrained mice (p=0.0368, 0.0095 ±0.0008) compared to saline group (p=0.0539 ±0.0057), while no significant change is observed in mice receiving IL-1β (p=0.0248 ±0.0116). When evaluating locomotion in the time domain across the 6-hour window, we observed a significant increase in inactivity time, indicative of sickness behavior in the TNF group (p=0.0003, 310.9 minutes ±8.23) compared to saline (216.9 minutes ±13.80), but not in the IL-1β group (271.5 minutes ±24.36). Taken together, these studies identify distinct physiological changes specific to IL-1β and TNF.

### P28. A rapid assay to detect choline acetyltransferase activity during inflammation

#### Arielle H. Gabalski^1,2^, Aisling Tynan^1^, Tea Tsaava^1^, Sam George^1^, Jian Hua Li^1^, Kevin J. Tracey^1,2^, Sangeeta S. Chavan^1,2^

##### ^1^Institute for Bioelectronic Medicine, The Feinstein Institutes for Medical Research, Northwell Health, 350 Community Drive, Manhasset, NY, USA; ^2^Donald and Barbara Zucker School of Medicine at Hofstra/Northwell, 500 Hofstra University, Hempstead, NY, USA

###### **Correspondence:** Sangeeta S. Chavan

Dysregulation of cytokine production is a hallmark of both acute and chronic inflammatory diseases, including endotoxemia and sepsis. We have previously established that acetylcholine (ACh), the classical neurotransmitter, suppresses inflammatory responses by binding to the alpha-7 nicotinic acetylcholine receptor expressed on cytokine-producing cells. Choline acetyltransferase (ChAT) is a rate-limiting enzyme that catalyzes acetylcholine synthesis. However, the temporal changes in ChAT activity during inflammatory disease conditions are not yet clear. Here, we developed a rapid and sensitive colorimetric assay to quantify ChAT activity in inflammatory conditions. Recombinant (rChAT) protein was incubated with known concentrations of ChAT substrates, choline and acetyl-CoA, followed by the colorimetric detection of the substrate depletion over time. A concentration-dependent change in the activity is observed using rChAT protein. Addition of alpha-NETA, a known ChAT inhibitor, blocks the enzyme activity of rChAT in a concentration-dependent manner. Kinetic analysis revealed a Michaelis-Menton constant (Km = 594.6 uM Choline) using a 2ug/mL concentration of rChAT. Next, we evaluated changes in ChAT activity during endotoxemia using this established assay. A significant increase in ChAT activity levels is observed beginning at 6 hours (Endotoxin: 6.422 ± 1.097 nmol/min/mg vs. vehicle: 3.903 ± 1.612 nmol/min/mg; p = 0.0092) till 48 hours (Endotoxin: 22.090 ± 7.852 nmol/min/mg vs. vehicle: 9.451 ± 3.486 nmol/min/mg; p = 0.0057), which return to baseline by 72 hours in mice challenged with bacterial endotoxin. Together, these studies indicate circulating ChAT as a novel target for inflammatory conditions.

### P29. Trigeminal nerve stimulation to modulate cortical spreading depolarizations after ischemic stroke

#### Daniel Lynch^1,2^, Keren Powell^1^, Kevin Shah^3^, Raj Narayan^1,3^, Chunyan Li^1,2,3^

##### ^1^Translational Brain Research Laboratory, The Feinstein Institutes for Medical Research, Manhasset, NY, USA; ^2^Zucker School of Medicine at Hofstra/Northwell, Hempstead, NY, USA; ^3^Department of Neurosurgery, North Shore University Hospital, Manhasset, NY, USA

###### **Correspondence:** Chunyan Li

Background: Cortical spreading depolarization (CSD) is a phenomenon of depressed electrical activity in the brain that has been clinically associated with a variety of acute brain injuries, including 100% of patients with malignant hemispheric ischemic stroke. In addition to being a real-time marker of brain damage, CSDs are also believed to be a mechanism of secondary injury in the compromised tissue of acute brain injuries. Thus, recent studies have focused on the use of various drugs to create complete cessation of CSDs in the injured brain as a method of preventing further tissue loss. However, these pharmacological approaches are systemic and typically have significant side effects. Therefore, new strategies are needed to selectively reduce the deleterious consequences of CSDs. The trigeminal nerve is the largest cranial nerve forming an extensive network throughout the central nervous system and is unique because of its intimate connection with cerebral and meningeal blood vessels, referred to as the trigemino-cerebrovascular system. Trigeminal nerve stimulation (TNS) is a noninvasive bioelectronic modality known to play a key role in cerebrovascular tone regulation.

Objective: To investigate the ability of TNS to decrease CSD generation in an ischemic stroke model

Methods: Studies were performed on 32 male Sprague-Dawley rats. Animals were randomized to four study groups in injured brain with middle cerebral artery occlusion (MCAO): (1) control animals with permanent MCAO; (2) MCAO animals with Pre-TNS (intermittent TNS for 60 min); (3) MCAO rats with Post-TNS (low dose); and (4) MCAO rats with Post-TNS (high dose). The TNS was triggered by inserting two bipolar wires bilaterally targeting the left infraorbital branch of the trigeminal nerve. Rectangular biphasic pulses (50 Hz, 0.5 ms) with amplitude of 0.25-5 V were delivered for 1 minute every 10 minutes and the dose of TNS was controlled by the stimulation voltage. Three epidural Ag-AgCl electrodes were implanted over the left hemisphere to record CSDs. At 24 h after MCAO, rats were sacrificed to measure the lesion volume.

Results: MCAO resulted in a sequence of changes in CBF and DC potentials. Upon occlusion, CBF immediately fell by 68 ±11%. Spontaneous waves of depolarization appeared in the ischemic penumbra zone, averaging about eight events (8.1 ±2.1; n=8) over the 3 h after occlusion. The first CSD episode appeared at 7.1 ±3.6 min after occlusion. In the MCAO rats, Pre-TNS treatment did not alter the amplitude or duration of each CSD. However, Pre-TNS treatment significantly lengthened the latency until the appearance of the first CSD almost 7-fold, and decreased their number by 53% (3.8 ±0.8 vs. 8.2 ±2.1; n=8). Pre-TNS treatment just before MCAO also significantly reduced infarction volumes by 34% (from 218.5 ±42.6 to 143.1 ±24.7 mm3; n=8). Both low and high dose of Post-TNS treatment also significantly reduced infarction volumes by 39% and 51%, respectively.

Conclusion: The results of our study demonstrate that TNS can selectively reduce the deleterious consequences of CSDs in the ischemic brain and decrease the lesion volume in a dose-dependent manner. The results suggest the therapeutic action of the TNS can salvage at-risk penumbra after ischemic stroke.

### P30. Natural and biomimicked diving reflex promote neuroprotection after chronic cerebral hypoperfusion

#### Willians Tambo^1,2^, Keren Powell^1^, Steven Wadolowski^1^, Raj K. Narayan^1,3^, Chunyan Li^1,2,3^

##### ^1^Translational Brain Research Laboratory, The Feinstein Institutes for Medical Research, Manhasset, NY, USA; ^2^Elmezzi Graduate School of Molecular Medicine, Manhasset, NY, USA; ^3^Department of Neurosurgery, North Shore University Hospital, Manhasset, NY, USA

###### **Correspondence:** Chunyan Li

Background: Chronic cerebral hypoperfusion (CCH) is a significant clinical entity in aging populations, especially patients with neurodegenerative diseases, such as Alzheimer's disease or Parkinson's disease. The main pathophysiological cascade in CCH involves oxidative stress and neuroinflammation in the hippocampus and white matter, leading to neurological and cognitive impairment. Despite its significance, there’s a limited therapeutic approach. It is necessary to develop new forms of treatment capable to ameliorate the long-term consequences of CCH. The diving reflex (DR) is a naturally occurring defensive mechanism mediated via the trigeminal nerve that geared towards surviving of hypoxic/anoxic conditions. It can be induced by cold water or noxious gases applied to the anterior nasal mucosa and paranasal regions, which stimulate the trigeminal thermo- or chemo-receptors to send afferent signals to medullary nuclei. Despite its significant therapeutic potential, it has yet to be adopted into routine clinical practice due to its yet-to-be-defined dose-response relationships, uncertain reproducibility, and lack of clinical studies for this indication. We have previously shown that electrical stimulation of the trigeminal nerve (TNS) can trigger specific physiological changes compatible with the pattern of the classic DR observed in animals/humans. This opens up the possibility of investigating its protective mechanisms against various insults in a well-controlled research setting. We hypothesize that both the natural and biomimicking DR induced by TNS could activate nuclear factor erythroid 2-related factor 2 (Nrf2) signaling pathway to target multifactorial mechanisms after CCH.

Objective: To investigate the ability of natural and biomimicked DR to promote neuroprotection after CCH through activation of Nrf2 signaling pathway

Methods: The permanent bilateral common carotid arteries occlusion (2VO) was performed to establish the CCH model. Male Sprague-Dawley rats were randomized to four study groups: (1) sham rats; (2) 2VO control rats; (3) 2VO rats with natural DR treatment (2VO+DR); (4) 2VO rats with TNS-induced DR treatment (2VO+TNS). For natural DR treatment group, rats were undergone to pre-DR training, and DR treatment were delivered at 3 days after 2VO. The rats were assessed for cognitive, behavioral, and sensorimotor impairment for 6 weeks. At 42 days, the rats were sacrificed for the analysis.

Results: Both natural DR and TNS-induced DR increased mRNA and nucleus protein levels of Nrf2 and key neuropeptides including calcitonin gene related peptide (CGRP) and pituitary adenylate cyclase activating polypeptide (PACAP). 2VO caused memory, anxiety, and sensorimotor dysfunction. However, 2VO+DR group significantly reduced memory and anxiety impairment. In addition, 2VO+DR significantly decreased neuronal pyknosis, cytotoxic and vasogenic edema in the hippocampus and amygdala.

Conclusion: Our experimental results show that both natural and biomimicking DR upregulated mRNA and protein levels of Nrf2. In 2VO model, natural DR treatment group exerted significant neuroprotection by decreasing neuronal apoptosis, vasogenic and cytotoxic edema in the hippocampus and amygdala after CCH. The DR, non-pharmacological and non-invasive approach, might result in a potential treatment method against various neurodegenerative diseases associated with CCH.

### P31. Trigeminal nerve stimulation mitigates delayed cerebral ischemia and behavioral deficits following subarachnoid hemorrhage

#### Keren Powell^1^, Kevin Shah^2^, Prashin Unadkat^2^, Raj Narayan^1,2^, Chunyan Li^1,2^

##### ^1^Translational Brain Research Laboratory, The Feinstein Institutes for Medical Research, Manhasset, NY, USA; ^2^Department of Neurosurgery, North Shore University Hospital, Manhasset, NY, USA

###### **Correspondence:** Chunyan Li

Background: Subarachnoid hemorrhage (SAH) is associated with high mortality rates and high morbidity in survivors. Delayed cerebral ischemia (DCI), a multifactorial disorder with a combination of large artery vasospasm and microcirculatory failure, is one of the most feared complications of SAH. With the failure of clinical studies that have targeted vasospasm to show benefit, recent attention has been redirected to other potential causes of DCI such as microvascular dysfunction, inflammation, thromboembolism and cortical spreading depolarizations. Despite tremendous efforts to date, nimodipine is currently the sole FDA-approved treatment for SAH patients and its benefits are marginal at best. The trigeminal nerve, which richly innervates the cerebrovasculature and forms the trigemino-cerebrovascular network, releases a multitude of vasoactive neuropeptides upon stimulation, including calcitonin gene-related peptide (CGRP). In addition to being one of the most powerful vasoactive agents discovered, CGRP has been shown to be angiogenic, anti-inflammatory, and anti-oxidative in ischemic conditions. Electrical trigeminal nerve stimulation (TNS), therefore, has the potential to ameliorate many of the processes leading to DCI, including micro-and-macrovascular dysfunction, cortical spreading depolarizations (CSD), neuroinflammation, oxidative stress, excitotoxity, and breakdown of the blood-brain barrier.

Objectives: To assess whether TNS mitigates DCI and behavioral deficits following SAH

Methods: Male Sprague-Dawley rats were randomly assigned to one of three groups, sham, SAH-control, and SAH-TNS. The endovascular puncture model was utilized to induce SAH on day 0, followed by KCl-induced cortical spreading depolarizations on Day 1, concurrent to treatment with TNS. Behavioral dysfunction was assessed on Days 2 and 3, followed by sample collection on Day 3. Multiple parameters were assessed, including cerebral lesion development, CSD frequency, cerebral blood flow (CBF), microthrombosis, cytotoxic and vasogenic edema, calcitonin gene-related peptide release, and sensorimotor and memory dysfunction.

Results: SAH induced the development of significant lesion across the ipsilateral hemisphere of the brain, alongside development of microthrombi and cytotoxic and vasogenic edema throughout the hippocampal, cortical, and thalamic regions of the brain. Comparatively, treatment with TNS resulted in a 48.8% decrease in lesion development, as well as a 75-100% decrease in the number of microthrombi/ROI, 14-32% decrease in cytotoxic edema/ROI, and 20-88% decrease in vasogenic edema, depending on the specific region of the brain. TNS treatment delivered at 24h after SAH resulted in a 53.4% decrease in the number of CSDs induced by 1M KCl application, as well as a 23.2% increase in CBF, relative to the SAH-control brains. Furthermore, the hyperemic response to CSD was attenuated in SAH-control brains, with only 30.1% of the CBF increase in sham brains, and TNS augmented it, with 56.1% of the CBF increase in sham brains. Additionally, TNS increased the cerebrospinal fluid CGRP levels by 89% compared to SAH-control animals and ameliorated memory and sensorimotor dysfunction.

Conclusion: Our results suggest that TNS exerts a powerful response across multiple levels of cerebral circulation, thus decreasing the development of DCI through reducing cerebral ischemia, tissue damage, and behavioral dysfunction. It is likely that this response is partially mediated via the focal release of CGRP and the preservation of neurovascular coupling.

### P32. Parkinson’s Disease state estimation for closed-loop deep brain stimulation

#### Daryl Lawrence^1,2^, Tanner Dixon^2,3^, Md Fahim Anjum^2,3^, Philip Starr^2,3^, Simon Little^2,3^

##### ^1^Department of Bioengineering, University of California Berkeley – University of California, San Francisco, USA; ^2^Weill Institute for Neurosciences, University of California, San Francisco, USA; ^3^School of Medicine, University of California, San Francisco, USA

###### **Correspondence:** Simon Little

Deep brain stimulation (DBS) is an effective treatment for individuals with Parkinson’s Disease (PD). Closed-loop DBS in particular is an adaptive paradigm which seeks to optimally vary stimulation parameters based on a patient’s real-time symptom severity. The experiments conducted for this study recruited a patient who was implanted bilaterally with deep brain pacemakers that stimulated his subthalamic nuclei (STN). The current amplitude of the continuous DBS administered was modulated, and the patient was asked to perform a set of prescribed movement tasks. We then employed unsupervised clustering methods to identify different symptomatic disease states using data collected solely from wearable smartwatches with embedded accelerometers and gyroscopes. Based on movement and symptom severity measurements, we characterized each state as being healthy, high-tremor or high-dyskinesia while the patient was stationary or moving. Future work will develop supervised learning models using neural data, collected from the implanted neurostimulators, with wearable symptom severity labels as therapeutic targets. This approach will then be implemented as a closed-loop DBS control policy, wherein stimulation is optimized to increase time spent in healthy states.

### P33. Targeted transcutaneous cervical spinal cord stimulation promotes upper limb recovery in spinal cord and peripheral nerve injury

#### Santosh Chandrasekaran^1^, Nikunj A Bhagat^1,9^, Richard Ramdeo^1^, Sadegh Ebrahimi^1^, Pawan D Sharma^2^, Doug G Griffin^7^, Adam Stein^6^, Susan J Harkema^2,3,4,5^ and Chad E Bouton^1,8^

##### ^1^Neural Bypass and Brain Computer Interface Laboratory, Institute of Bioelectronic Medicine, Feinstein Institutes for Medical Research, Northwell Health, Manhasset, NY, USA; ^2^Kentucky Spinal Cord Injury Research Center, University of Louisville, Louisville, Kentucky, USA; ^3^Department of Bioengineering, University of Louisville, Louisville, Kentucky, USA; ^4^Frazier Rehabilitation Institute, University of Louisville Health, Louisville, Kentucky, USA; ^5^Department of Neurological Surgery, University of Louisville, Louisville, Kentucky, USA; ^6^Department of Physical Medicine and Rehabilitation, Donald and Barbara Zucker School of Medicine at Hofstra, Northwell Health, Manhasset, NY, USA; ^7^Northwell Health STARS Rehabilitation, East Meadow, NY, USA; ^8^Donald and Barbara Zucker School of Medicine at Hofstra/Northwell, Manhasset, NY, USA; ^9^Department of Physical Medicine & Rehabilitation, University of Texas Health Science Center, Houston, Texas, USA

###### **Correspondence:** Santosh Chandrasekaran & Chad E Bouton

Long-term recovery of limb function is a significant unmet need in people with paralysis. Targeted neuromodulation through epidural stimulation of the spinal cord, when paired with intense physical therapy, has shown promising results towards restoring volitional limb control in people with spinal cord injury. Non-invasive neuromodulation of the cervical spinal cord using transcutaneous spinal cord stimulation (tSCS) has shown similar improvements in upper-limb motor control rehabilitation. However, the potential rehabilitative effects of activating specific cervical spinal segments in a targeted fashion using tSCS has largely remained unexplored. We show in two individuals with SCI that tSCS of the cervical spinal cord resulted in up to 1136% increase in exerted force, with minimal physical therapy. We also show, for the first time, the effectiveness of targeted tSCS in restoring strength (407% increase) and dexterity to the digits in an individual with paralysis of the hand due to a peripheral nerve injury. Furthermore, we believe this is the first study to document a 3-point improvement in sensation in people with SCI or peripheral injury after receiving tSCS. Lastly, participant gains persisted after a one-month period void of stimulation, suggesting tSCS may lead to lasting benefits. Non-invasive targeted spinal cord stimulation shows tremendous promise as a safe and effective therapeutic approach with broad applications.

### P36. Threshold adjusted vagus nerve stimulation after asphyxial cardiac arrest results in reduction of calcium overload and apoptosis

#### Rishabh C. Choudhary^1,2^, Umair Ahmed^2^, Abdul Rehman^1^, Stavros Zanos^2^, and Lance B Becker^1,2,3^

##### ^1^Laboratory for Critical Care Physiology, Feinstein Institutes for Medical Research, Northwell Health, Manhasset, NY, USA; ^2^ Institute of Bioelectronic Medicine, Feinstein Institutes for Medical Research, Manhasset, NY, USA; ^3^ Department of Emergency Medicine, Northwell Health, NY, USA

###### **Correspondence:** Lance B Becker (lance.becker@northwell.edu)

Background: Brain injury caused by cardiac arrest (CA) is one of the major events of mortality. After CA, the brain undergoes delayed neurodegeneration, beginning from hours to days due to ischemic reperfusion injury. Several pathways play a foremost role in neuronal cell death after global brain ischemia, including excitotoxicity, mitochondrial dysfunction, endoplasmic reticulum stress, oxidative stress, inflammation, and dysregulation of intracellular Ca^2+^ homeostasis. The therapeutic role of vagus nerve stimulation (VNS) has been shown to improve survival with lower brain injury by reducing inflammation, and mitochondrial dysfunction, and improving cerebral blood flow. Although the therapeutic effect of VNS has been discussed, there is still a missing gap in understanding the cellular and subcellular mechanisms of VNS in reducing post-CA injury. Dysregulated intracellular Ca^2+^ homeostasis after cardiac arrest also contributes to cell death and is an underexplored area.

Objective: This study aimed to investigate the protective effects of threshold-adjusted VNS (tVNS) in a rat model post-CA in reducing intracellular Ca^2+^ overload and cell apoptosis.

Methods and Results: Male Sprague-Dawley rats underwent 12 min asphyxial-CA followed by resuscitation. Rats were assigned to either post-resuscitation tVNS for 2 h or no-tVNS (control). tVNS was applied by electrode placement on the left cervical vagus nerve immediately after the return of spontaneous circulation. The tVNS was determined by a 15-20% reduction from the immediate baseline heart rate as the effective and physiological threshold for each animal. This enabled customized parameters for individual animals. At 2 h post-ROSC, rats were perfused with normal saline, and the whole brain was removed. The whole brain was then processed for protein expression by western blotting. We also prepared sham rats that did not receive CA or tVNS. For measuring intracellular Ca^2+^ overload we evaluated the expression of Calpain-2 protein and for apoptosis, we measured the BCL2-associated agonist of cell death (BAD) protein phosphorylation (p-BAD). Calpain-2 is activated when the level of intracellular Ca^2+^ reaches a supraphysiological concentration whereas higher phosphorylation of BAD leads to less apoptosis. We observed a significantly higher protein expression of calpain-2 in whole brain tissue after CA in control groups, in comparison to the sham and tVNS groups (p<0.01). A significant increase in de-phosphorylation of p-BAD protein was observed after CA in the control group compared with sham (p<0.05), and no significant increase in de-phosphorylation of p-BAD protein was observed between the tVNS and sham groups.

Conclusion: The application of tVNS immediately after 12 minutes of CA and resuscitation results in reduced calpain-2 level and apoptosis. This data suggests that the protective mechanism of tVNS on cell apoptosis involves a reduction in intracellular Ca^2+^ overload, signifying, that tVNS may be a novel therapeutic approach in neuroprotection after CA.

### P37. Optogenetic activation of cholinergic neurons in the brainstem dorsal motor nucleus ameliorates acute pancreatitis

#### Dane Thompson^1,2,3,4^, Tea Tsaava^1^, Kevin J. Tracey^1,2,4^, Sangeeta S. Chavan^1,2,4^

##### ^1^Institute for Bioelectronic Medicine, Feinstein Institutes for Medical Research, Northwell Health, 350 Community Drive, Manhasset, NY 11030, USA; ^2^Donald and Barbara Zucker School of Medicine at Hofstra/Northwell, 500 Hofstra University, Hempstead, NY 11549, USA; ^3^Department of Surgery, Northshore University Hospital, Northwell Health, 300 Community Drive, Manhasset, NY 11030, USA; ^4^Elmezzi Graduate School of Molecular Medicine, 350 Community Drive, Manhasset, NY 11030, USA

###### **Correspondence:** Sangeeta S. Chavan

The nervous system regulates immunity and inflammation. The best studied anti-inflammatory neural pathway is the cholinergic anti-inflammatory pathway, where cholinergic neurons originating in the dorsal motor nucleus (DMN) control immune system responses by inhibiting the production of cytokines in the spleen. However, the function of DMN neurons in regulating pancreatic inflammation is not known. Here, we used optogenetics to selectively activate cholinergic nerve fibers within the DMN and evaluated the effects on caerulein-induced pancreatitis. Pancreatitis was induced with two intraperitoneal injection of caerulein (50 mcg/kg), one hour apart. A fiber-optic probe was inserted, under stereotactic guidance, into the brainstem of ChAT-ChR2 mice which express channel rhodopsin (ChR2) on choline acetyltransferase-positive (ChAT) cholinergic neurons. Optogenetic activation but not sham stimulation of DMN cholinergic neurons significantly reduces levels of serum amylase (sham vs stimulation: 4043 ± 877 vs 2940 ± 512.8 mU/mL, p = 0.0164; n = 9-11 mice/group), pancreatic IL-6 (1907 ± 879.3 vs 1046 ± 288.1 pg/mg, p=0.033), and pancreatic monocyte chemoattractant protein-1 (MCP-1) (953.1 ± 461.8 vs 471.2 ± 241.7 pg/mg, p = 0.0123). Accordingly, a significant decrease in pancreatic histological severity, including reduced edema (sham vs stimulation: 2.7 ± 0.7 vs 1.8 ± 0.8, p = 0.05, n = 9-11) and overall reduced histological severity (sham vs stimulation: 7.2 ± 1.2 vs 5.45 ± 1.4, p = 0.01, n = 9-11) is observed. Subdiaphragmatic vagotomy abolishes the protective effects of DMN cholinergic stimulation (sham vs stimulation: 2761 ± 787.3 vs 2517 ± 746.7 mU/mL, p = 0.30; n = 3). Pharmacological inhibition of nicotinic acetylcholine receptors with mecamylamine also blocks the protective effects of DMN stimulation (Sham vs stimulation vs stimulation + mecamylamine: 5047 ± 315.4 vs 3769 ± 241.5 vs 5620 ± 385.9, p = 0037; n = 10 mice/group). These observations establish that stimulation of cholinergic neurons in the DMN is sufficient to provide pancreatic protection and encourage further evaluation as a potential disease preventing therapy for acute pancreatitis.

### P38. Synovial fluid mesenchymal stem cell-derived exosomes combined with platelet-rich plasma reduce inflammatory gene expression in chondrocytes and promote anabolic activity

#### Rhea Rasquinha, Pooja Swami, Haixiang Liang, Henintsoa Fanjaniaina, Daniel A. Grande

##### Orthopedic Research Laboratory, The Feinstein Institutes for Medical Research, Northwell Health Manhasset, NY 11030 USA

###### **Correspondence:** Daniel A. Grande

Introduction: Platelet-rich plasma (PRP) has been found to stimulate cell ECM production and mitigate inflammation in vitro and reduce knee pain in clinical trials. However, the efficacy of PRP therapy is limited due to variability in PRP preparation, heterogeneity among studies, platelet concentrations, application frequency and rehabilitation regimens. Platelets in PRP can also be a source of pro-inflammatory cytokines and other molecules, contributing to pain and swelling after intra-articular injection. Exosomes are small extracellular vesicles carrying complex microRNA and proteins that have potential to alleviate cartilage degradation and pain. In prior studies, exosomes derived from synovial fluid mesenchymal stem cells (SF-MSCs) were found to promote expression of ECM proteins and reduce inflammation and cartilage damage. The purpose of this study is to investigate the combined effect of PRP and SF-MSC derived exosomes on pro-inflammatory, catabolic, and anabolic gene expression. We hypothesized that addition of SF-MSC exosomes would reduce the expression of inflammatory related genes post PRP treatment.

Methods: Exosomes were collected from SF-MSC prior to experimentation and particle number was determined using the ZetaView NTA. PRP was collected briefly prior to treatment using the Emcyte kit and platelet count was assessed using the Horiba Hematology Analyzer. Human chondrocytes were plated in 12 well plates and were divided into five groups, four of which were stimulated with IL-1β (10ng/ml) and Oncostatin M (20ng/ml) to induce inflammation. The experimental groups were inflammation only (OA/positive control), PRP (5% v/v), Exo (4.4*1010 particles/well), P+E (PRP + Exosomes 4.4*1010 particles/well). A negative control (NC) group did not receive cytokine stimulation. After 24 and 96 hours, chondrocytes were lysed for mRNA isolation and cDNA synthesis. qPCR was performed to analyze the relative gene expression of TNF-α, ADAMTS4, MMP-3, MMP-13, ACAN, PCNA, PRG4, and SOX9. Comparisons between group pairs were analyzed with student’s two-tailed t-tests.

Results: The gene expression was consistent with the expected changes between the negative and the positive OA control, validating the OA model. There was a significant increase in TNF-α and ADAMTS4 expression on D1 in PRP treated cells compared to the positive control. There was a significant decrease in MMP-3 expression at both time points and a significant increase in SOX9 expression on D1in the PRP alone group compared to the positive control. The combined P+E group had significantly lower expression of TNF-α at both time points and MMP-13 on D1, and lower expression in ADAMTS4 and MMP-3, but did not reach statistical significance compared to PRP alone. On D4, there was a significant increase in PRG4 expression in the combined group compared to PRP alone, along with increases in ACAN, PCNA and SOX9 expression. MMP-3 expression was significantly decreased in the combined P+E group compared to the positive control at both time points. There was a significant increase in ADAMTS4 expression in the combined group on D1, but expression was significantly lower on D4 compared to the positive control. ACAN expression was significantly decreased and PRG4 expression was lower on D1 in the combined group compared to the positive control (p<0.05, =0.260, respectively), but expression increased on D4 and was significant for PRG4 (p=0.22, <0.01, respectively). SOX9 expression was also higher in the combined group on D1 (p=0.053).

Discussion and Conclusion: This data demonstrates that PRP alone both causes a pro-inflammatory response and promotes proliferation and some protection of the ECM in the short term. Pro-inflammatory effects of PRP were observed only at the earlier time point post-administration, and protective effects from the added exosomes were observed at the later time point. Combining PRP with SF-MSC-derived exosomes mediates this early inflammation and shows an increase in anabolic markers at the later time point. This suggests that exosomes could be a beneficial addition to PRP treatment and improve long term outcomes.

Significance: To our knowledge, this study is the first to report on the effects of combining PRP and exosomes in vitro in a chondrocyte inflammatory model. Thus, our study provides information on the effects of PRP and exosomes on joint inflammation and presents a new agent to decrease the side effects of PRP treatment.

### P39. Development and application of implantable wireless neuromodulation systems for small animal research

#### Jason Wong, Jose Mathew, Mohamed Elgohary, Ibrahim Mughrabi, Tea Tsaava, Tyler Hepler, Timir Datta-Chaudhuri

##### Institute of Bioelectronic Medicine, The Feinstein Institutes for Medical Research, Northwell Health, 350 Community Drive, Manhasset, NY, USA

###### **Correspondence:** Timir Datta-Chaudhuri

Mice are the most commonly used animal model in medical research due to their ease of care, shorter lifespans, availability of transgenic strains, and range of established disease models. Particularly in the field of bioelectronic medicine, research technologies used for neuromodulation in small animals need improvement. Chronic animal experiments often require tethering the animal to benchtop equipment via a headstage in order to deliver stimulation or gather recordings; anesthetization may also be required, impacting the animal’s physiological response to stimuli. Currently available systems have limited feature sets (e.g. stimulation only or recording only capabilities, lack of wireless charging) due to the challenge of miniaturization and packaging of electronics in a form factor suitable for chronic implantation. The development of several generations of small animal neuromodulation systems are described here showing progress towards a fully wireless system capable of neural stimulation and recording, wireless charging, and a bidirectional wireless interface for control of stimulation parameters and readout of physiological signals. Animal data shows successful chronic implantation in mice without negative impacts on animal health or behavior. Vagus nerve stimulation (VNS) was performed in chronically implanted mouse models of endotoxemia. Mice receiving regular administration of VNS prior to peritoneal injection of lipopolysaccharide showed significantly lower levels of serum TNF when compared to sham animals.

